# Wider Than the Sky: An Alternative to “Mapping” the World Onto the Brain

**DOI:** 10.1111/ejn.70224

**Published:** 2025-08-26

**Authors:** Ann‐Sophie Barwich, Stuart J. Firestein, Michael R. Dietrich

**Affiliations:** ^1^ Department of History and Philosophy of Science and Medicine Indiana University Bloomington Bloomington Indiana USA; ^2^ Cognitive Science Program Indiana University Bloomington Bloomington Indiana USA; ^3^ Program in Neuroscience Indiana University Bloomington Bloomington Indiana USA; ^4^ Department of Biological Sciences Columbia University in the City of New York New York New York USA; ^5^ Department of History and Philosophy of Science University of Pittsburgh Pittsburgh Pennsylvania USA

**Keywords:** genetic transcription, morphological computation, olfaction, philosophy of neuroscience, pluralism, representational drift, sensory coding, topographic paradigm

## Abstract

This paper reevaluates the conventional topographic model of brain function, stressing the critical role of philosophical inquiry in neuroscience. Since the 1930s, pioneering studies by Penfield and subsequent advancements in visual neuroscience by Hubel and Wiesel have popularized the concept of cortical maps as representations of external and internal states. Yet contemporary research in various sensory systems, including visual cortices in certain animals, questions the universal applicability of this model. We critique the restrictive influence of this paradigm and introduce an alternative conceptualization using the olfactory system as a model. This system's genetic diversity and dynamic neural encoding serve as a foundation for proposing a rule‐based, adaptive framework for neural processing, akin to the dynamic routing in GPS technology, which moves beyond fixed spatial mappings.

## Graphical abstract

- Challenging the topographic paradigm: The olfactory system's dynamic organization and phenomena like representational drift challenge traditional spatial mapping models, advocating for flexible, rule‐based frameworks in neuroscience.
- Dynamic, context‐dependent encoding: Experience‐dependent plasticity and morphological computation in olfaction illustrate how neural systems adapt to environmental stimuli, offering insights into flexible sensory processing across modalities. Leveraging genetic transcription and neurogenesis, the olfactory system enables adaptive sensory memory and information patterning without static spatial representations.

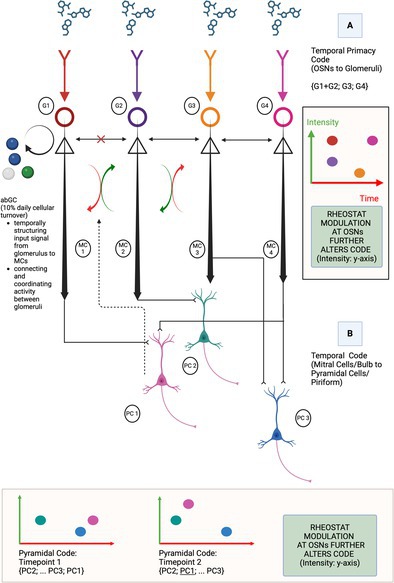

**Abbreviations**
abGC: adult‐born granule cells
AON: anterior olfactory nucleus
cAMP: cyclic adenosine monophosphate
CREB: cAMP response element‐binding protein
FFA: fusiform face area
GPCR: G‐protein–coupled receptor
GPS: Global Positioning System
LTD: long‐term depression
LTP: long‐term potentiation
MC: mitral cell
M/T: cells mitral/tufted cells
OR: olfactory receptor
OSN: olfactory sensory neuron
Pax6: paired box protein
PC: pyramidal cell
RNA: ribonucleic acid
SOX2: SRY (sex‐determining region Y)‐box 2
STDP: spike‐timing–dependent plasticity
V1: primary visual cortex (striatum)
V2: visual area V2/secondary visual cortex (prestriate cortex)
V4: visual area V4 (extrastriate visual cortex)

The Brain—is wider than the Sky—
For—put them side by side—
The one the other will contain
With ease—and you—beside—
(Emily Dickinson, 1862)

## 1 Introduction

In the late 1930s, Wilder Penfield discovered during neurosurgeries that stimulating small portions of the brain would elicit characteristic responses in patients. In 1937 and 1950, Penfield published his now‐famous homunculus, which would become an iconic symbol of brain architecture (Penfield and Boldrey 1937; Penfield and Rasmussen 1950). This was the beginning of an idea about brain architecture that has dominated neuroscience research, to the nearly complete exclusion of all other possibilities—at least until the last few years. Work on cortical columns and visual representation by Vernon Mountcastle (Mountcastle et al. 1957), David Hubel and Torsten Wiesel (1959, 2004)—awarded a Nobel prize for their work in 1981—and their later computational counterpart David Marr (1982) gave further support to this idea of a neural “representation” of the world mapped onto brain space. These influential reports further propelled this research program (Shepherd 2009; Haueis 2016), which now involves many laboratories and thousands of postdocs and graduate students, using increasingly refined neuroimaging techniques on genetically engineered and highly homogeneous model organisms. Even the occasional inexplicable result—for example, many animal cortices do not possess a columnar organization (Naumann et al. 2015; Laurent et al. 2016; Fournier et al. 2018)—has not slowed the research effort or diminished the idea that the brain represents the outside world by constructing a map in three‐dimensional neural space.

Lurking just below the surface, and carefully sidestepped by the experimental community, is the troubling question of who is actually making sense of such a cortical roadmap. Is there a tiny executive homunculus watching the visual, acoustic, somatosensory, and other sensory maps and sending out instructions to muscles in accordance with the picture of the world it is receiving? Surely, no one subscribes to this; the homunculus appears as an ancient and silly idea today (Dennett 1993). But then, who *is* reading the map? Or why construct a map if we discard its reader?

This is a profoundly philosophical question, and ignoring it has led to years of scientific effort chasing down a singular model that now seems to be in need of substantial revision, assuming it is worth preserving at all. It is one example of the perils of ignoring philosophical questions when they might upset an admittedly large cache of experimental data. It is also a cautionary tale about the severely monistic approach modern science takes to many of its most fundamental problems. Other alternatives were and are available as possible models. Data from other than the predominant systems (cats, monkeys, humans) could have suggested alternative models that were never imagined, let alone ignored. Instead, the work of Hubel and Wiesel and the Nobel committee's recognition directed brain research along an overly narrow path for over 50 years.

This paper exemplifies the fecundity of philosophical analysis in current neuroscience by demonstrating how it can lead to a deeper and alternative understanding of brain function, promoting a shift away from rigid paradigms toward a more pluralistic and integrative approach in neuroscience. Our argument unfolds as follows: We start by delineating the kernel of the topographic paradigm in its origin, implications, and challenges. Section 2 examines the historical development of the topographic model in neuroscience, influenced by the work of Hubel and Wiesel. Section 3 explores the limitations of this paradigm, considering findings in the olfactory system, which lacks stereotypic spatial organization and shows dynamic, experience‐dependent neural encoding. Next, we examine why olfaction serves as a valuable model system for neuroscience, highlighting its potential to provide a robust framework for developing an alternative model of sensory encoding. Drawing on philosophical discussions regarding the genesis of scientific knowledge, Section 4 discusses how olfaction can significantly inform general neuroscience. Section 5 puts this theoretical argument into practice with an alternate account of sensory information encoding, as guided by genetic transcription mechanisms and driven by neurogenesis that modify responses according to environmental and experiential factors. This account demonstrates how sensory systems employ rule‐based mechanisms to process data dynamically, eliminating the requirement for spatial maps or static neural representations. Against this backdrop, we conclude with the implications of olfactory research for other sensory systems, urging the development of a more flexible paradigm in neuroscience. This approach should aim for broad applicability while carefully accommodating the distinct characteristics of different systems shaped by diverse evolutionary pressures, thereby enriching our holistic understanding of neural function.

## 2 Historical Background: The “Visualization” of Modern Neuroscience

The prevailing model of sensory cortices presents us with a blueprint of a beautifully systematic correlation between sensory inputs and the hierarchical, spatial patterning of neural activity. This principle has delineated the paths of vision and audition with deceitful clarity. How does the brain translate a raw cacophony of light and other sensory inputs from the external world into a coherent perceptual narrative? This question monopolized neuroscientific discourse for most of the 20th century (Shepherd 2009). Sensory systems, as complex networks of cells, capture the world in patterns both spatial and temporal, creating perceptual imagery from the language of neural firings.

In the late 1950s, a serendipitous discovery promised to shed light on this issue. Hubel and Wiesel (1959, 1960, 1961, 1962, 1963, 1965, 1968), postdocs in Stephen Kuffler's (1953) laboratory, embarked on a series of experiments involving the visual cortex. Their initial forays, monitored through microelectrodes delicately placed in the V1 region of the cat cortex, yielded a striking finding: It was not just any stimulus that these cells responded to, but lines, particularly lines in specific angles and orientations. Neurons, with approximate responses to visual input, cluster together, creating a cellular map of preferences and inclinations. Hubel and Wiesel's (2004) research suggested that visual system processing is a hierarchically coded reconstruction of input, a complex computation performed by the neural apparatus.

These findings did not arise in a vacuum. Hubel and Wiesel's work on the visual cortex built directly on Mountcastle's work on the columnar structure of the somatosensory cortex. Yet Mountcastle (1957, 432) was careful to emphasize that his observations reflected fundamental properties of cellular input and connectivity: “The neurons encountered in a perpendicular traverse of the cortex are activated from almost identical peripheral receptive fields at latencies which on the average are not a function of the position of the cell in depth within the cortex.” Once Hubel and Wiesel extended the columnar framework to vision, however, an important question lurking in Mountcastle's analysis was soon forgotten: How do systems with nonidentical receptive fields (such as the olfactory system, discussed in Section 3) fit into this framework?

**FIGURE 1:**
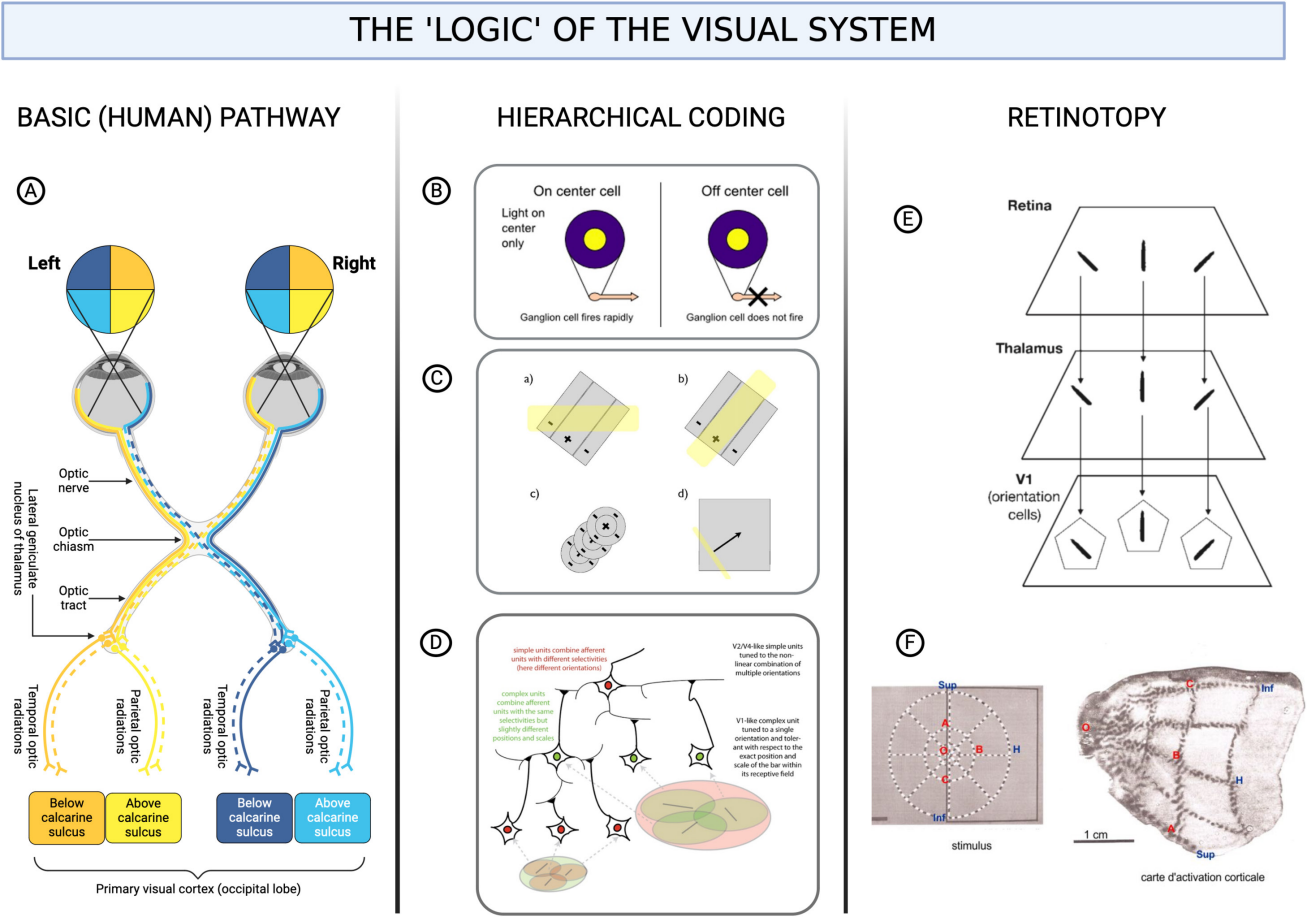
(Created in BioRender, Barwich 2025, https://BioRender.com/h39n006): The “logic” of the visual system after Hubel and Wiesel. (A) Schematic anatomy of the (human) visual pathway from the retina to the thalamus to the striatum. (B) Kuffler's center surround (on and off) cells detecting contrast by being activated when light hits the center of their receptive field and inhibited when light hits the surrounding area or vice versa (image: delldot and Xoneca 2009). (C) Receptive field of a neuron in the V1, hierarchically integrating signals from the retina and LGN/thalamus (image: Kyle.wg3139 2013). (D) Receptive field integration: hierarchical processing of visual information through increasingly complex “representations” of neuronal input at successive levels of the visual system (image: Serre‐lab, NA n.d.). (E) Extremely simplified principle of retinotopy: mapping of visual input from the retina to corresponding locations in the visual cortex, preserving the spatial organization of the visual scene (image: Barwich 2020a). (F) Tootell et al.'s (1988) detailed map of the visual striatum highlighting the retinotopic organization of visual field representations in the cortex (image: Pankrat 2011).

In the wake of Hubel and Wiesel, neuroscience experienced a paradigm shift that can be attributed to two reasons. First, their model of cortical processing began to unify an assortment of disparate single‐cell recordings into a coherent whole. Instead of tracking signals from Cell A to their projection in Cell B, these studies indicated the design through which the visual system transmuted raw stimulus data into three‐dimensional objects (Hubel 1988). Their findings revealed that visual representations were not the result of a homogeneous contribution from individual neurons, as had previously been assumed (e.g., McCulloch and Pitts 1943; Piccinini 2004), but specialized clusters of cells working together. Second, their research laid the groundwork for a methodology that successfully directed future investigations. This hierarchical, nested paradigm of visual processing—occasionally stretched to its limits (as evidenced by instances such as grandmother cells; Barwich 2019)—provided a fresh perspective on the structure of the brain, prompting Marr (1982) to contemplate the exact computations it carries out and inspiring his influential three‐stage model of visual object construction (Bickle 2015). It was difficult not to be enamored by the apparent logic of the visual system (Figure 1).

It seemed almost inevitable that this reasoning would prevail following Tootell et al. (1988). By employing radioactive glucose, they investigated metabolic activity in the striate cortex of monkeys, observing how different areas of this region responded to specific segments of the visual field. A critical element emerged: retinotopic mapping, where the visual cortex mirrors specific areas of the retina, creating a precise correspondence between the origin of visual signals in retinal cells and their cortical destination.

Research on other sensory systems, specifically audition, initially echoed these findings in vision, showcasing a comparable organization (Chittka and Brockmann 2005). The tacit assumption that guided models of cortical maps across sensory systems is that certain brain regions or cell populations consistently exhibit patterned responses to environmental stimuli. The topographic paradigm in neuroscience was cemented.

Revolutions, once they dominate the discourse, can end up in tyranny, though. Science, as we know, does not stick to a script (Medawar 1963; Schickore 2008; Firestein 2012, 2015), and brain research soon revealed unexpected complications beneath ostensibly orderly maps.

Neural processing is not a one‐way street. For example, recent insights into the motor strip and auditory cortex cast doubt on the tidy models of topographic organization. Finding multiple body mappings within the motor strip (Gordon et al. 2023) and a columnar architecture of the auditory cortex distinct from that observed in visual or somatosensory systems (Linden and Schreiner 2003) invites reassessments of the old paradigm. These studies are not isolated incidents but emerge as signs of a larger need to reevaluate topography as the primary organizational principle governing neural activity, with cracks beginning to show also in the conventional model of vision (Livingstone et al. 2017). Modern vision research reveals a highly complex picture of sensory processing, far surpassing its foundational heritage. Non‐topographic models for sensory neuroscience are thus gaining interest (Rayner 1998; Spivey 2008; Tanenhaus et al. 1995).

We contend that incorporating insights from olfaction, a model system that has been overlooked until now, could greatly enhance current trends toward a reevaluation of sensory coding.

## 3 Contemporary Developments: Neuroscience “Olfactorized”

The sense of smell presents an intriguing challenge to conventional approaches in neuroscience, most notably the topographic paradigm. While olfactory signaling appeared to follow a pattern similar to a well‐organized stimulus‐feature map, a markedly different picture emerges upon closer inspection.

**FIGURE 2:**
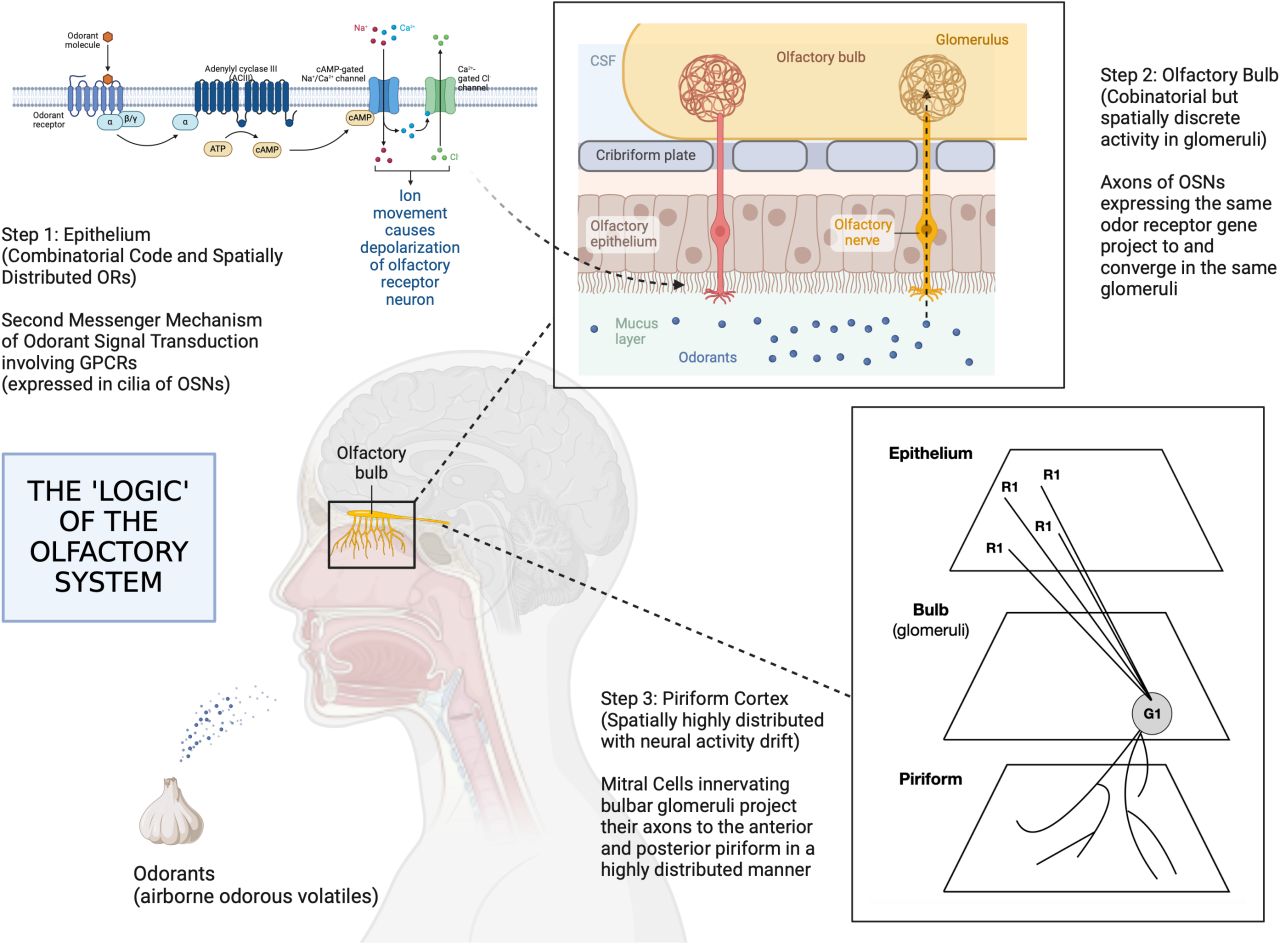
(Created in BioRender, Barwich 2025, https://BioRender.com/b58m556): Basic three‐level route of the olfactory pathway. The olfactory pathway begins with odorant molecules binding to receptors in the nasal epithelium, sending signals via the olfactory nerve to the olfactory bulb. From the olfactory bulb, the signals are relayed to the olfactory cortex and other brain regions such as the amygdala and hippocampus for integration, processing, and perception of smells. (Image in Step 3: Barwich 2020a) Not depicted is the intricate circuitry of granule cells, mitral cells, and various types of interneurons in the olfactory bulb, creating complex processing patterns of excitation and inhibition in the olfactory bulb (see Shepherd and Greer 1998; Shepherd et al. 2007; Kay and Murray Sherman 2007; Shepherd et al. 2021).

From a broader view, the olfactory pathway presents a *deceptively* shallow three‐level route from the air to the cortical core (Firestein 2001): Two synapses connect epithelial sensory neurons to the piriform, the largest area of the primary olfactory cortex (Figure 2). Odor processing kicks off with the molecular receptors, olfactory GPCRs—the largest multigene family in the mammalian genome—expressed in the cilia of sensory neurons (Buck and Axel 1991; Shepherd et al. 1996; Firestein 2005; Kurian et al. 2021). Upon encountering a wide variety of chemical structures, this interaction triggers signals that are then sent to and organized within the glomeruli of the olfactory bulb (Singer et al. 1995; Mombaerts et al. 1996), located in the inferior frontal lobe. The bulb displays, or appears to display, a unique activation pattern for every odorant, like an olfactory fingerprint, in contrast to the spatially widely dispersed receptors in the epithelium (Shepherd 2012; Lodovichi 2021). Following the lead of the visual system, it was assumed that such spatially discrete activity in response to odorants within the bulb would persist into the piriform cortex, possibly beyond.

But this notion has been radically upended in the last decade, and it matters why that is the case (Barwich 2020a). Three factors are at play here: the encoding of stimulus properties at the receptor sheet, the genetic and evolutionary‐developmental basis of the olfactory bulb, and the phenomenon of “representational drift” found in the piriform cortex. Taken together, these factors compel us to reconsider the topographic principle adopted from vision.

### 3.1 Patterns in the Olfactory Bulb Are Not Topographic

We must begin with the bulb. The seemingly orderly spatial arrangement of odor signals suggested that a topographic principle might govern odor processing, much like retinotopy in vision or tonotopy in hearing (Sharp et al. 1975; Mori and Yoshihara 1995; Xu et al. 2000; Uchida et al. 2000; Mori et al. 2006). Yet this initial assumption—that the olfactory bulb organizes odors or its physicochemical input through a stereotypic, topographic scheme—calls for a thorough reevaluation (Zou et al. 2009). This is largely because the arrangement of glomeruli is neither predictable nor static, but dynamic, diverging markedly from a rigid genetic blueprint.

Here, we highlight key features of the olfactory bulb that call for a reconsideration of its functional organization: glomerular receptive field tuning, developmental wiring, and local synaptic architecture. A fourth feature—neurogenesis—adds another layer of complexity and will be discussed in Section 5.

#### 3.1.1 The Argument From Function

Consider first *the argument from function*: An explication of what glomerular activity actually “represents” quickly dashes hopes for topographic models in olfaction. When we consider the functionality of the olfactory bulb, we might initially expect each glomerulus to serve as a clear‐cut representation of specific odor receptors (ORs), much like pins on a map. Each glomerulus acts as a converging point for the axonal projections of sensory neurons, which typically express a single OR gene (Mombaerts et al. 1996). This arrangement indicated that glomeruli reflect the activity of their corresponding receptors, following the widely held “one gene–one neuron” doctrine,^1^ suggesting a clear, structured odor activity map in the bulb. This neat arrangement would certainly simplify the brain's daunting task of decoding smells. Nevertheless, this idea might be more of a logician's wishful thinking than neurobiological reality.

**FIGURE 3:**
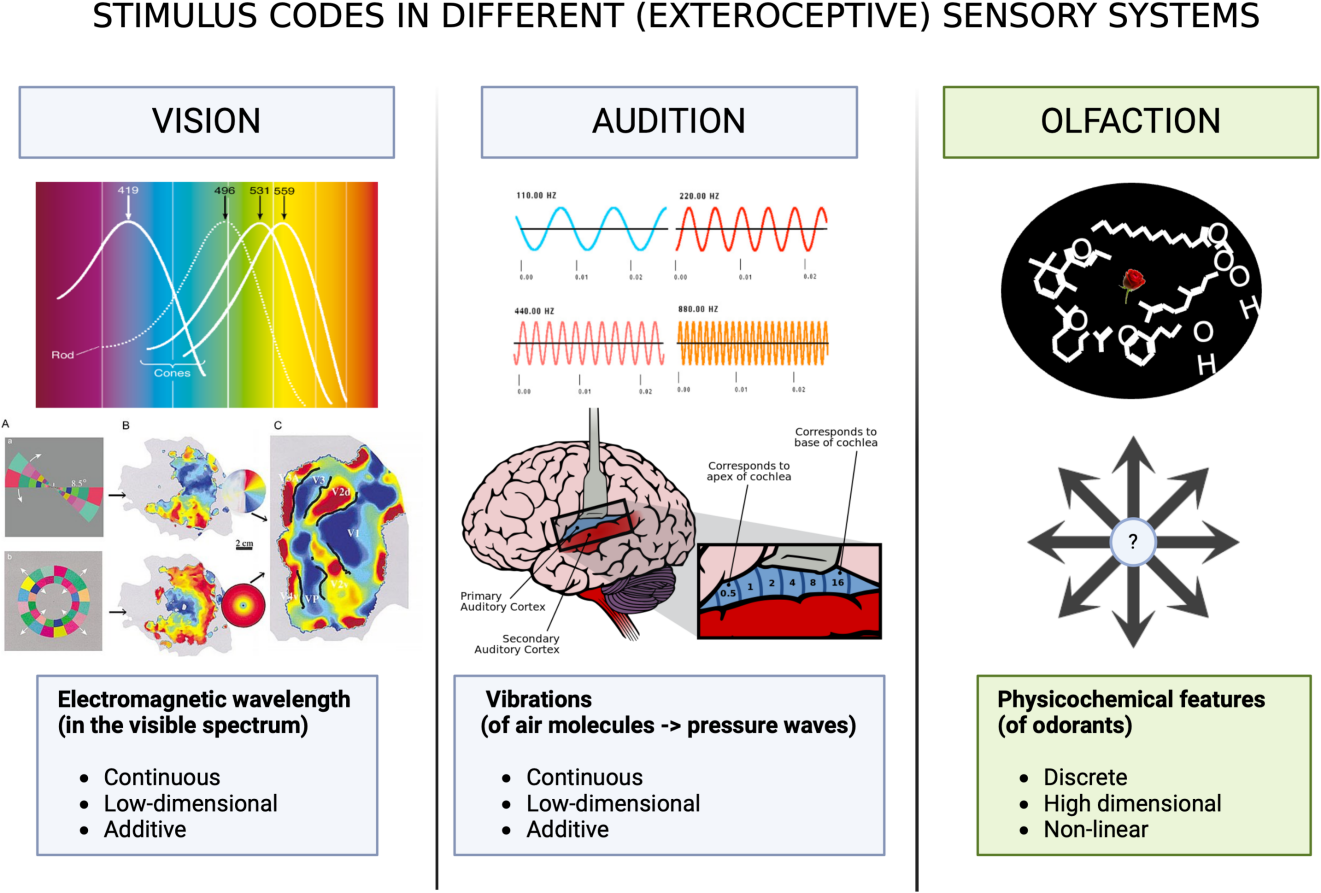
(Created in BioRender, Barwich 2025, https://BioRender.com/g74l844): Stimulus codes in different exteroceptive sensory systems. Left: Color vision involves electromagnetic wavelengths in the visible spectrum, mapped onto neural space via retinotopy (bottom image: Wikimedia, LordFarkquaad 2013). Middle: Audition processes air molecule vibrations (pressure waves), mapped onto neural space via tonotopy (bottom image: Chittka and Brokmann 2005). Right: Olfaction deals with discrete and nonlinear physicochemical features of odorants, which are not mapped onto neural space through a similar principle like “chemotopy” or “odotopy” (top image: Firestein).

A crucial issue with the notion of topographic stimulus representation in olfaction pertains to the constitution of the distal stimulus itself. In contrast to the low‐dimensional continuums of vision and audition, the olfactory system engages with a complex array of high‐dimensional stimuli (Figure 3). Like color receptors, ORs exhibit graded sensitivity—just to chemical features rather than wavelengths. Yet such initial resemblance masks a profound difference. Unlike the neatly segmented spectrum of light processed by retinal cones, “odorants” are not uniformly segmented or arrangeable in sequential physical chunks like the specific ranges of electromagnetic wavelengths processed by the retinal cones. Instead, odorants comprise thousands of structurally highly diverse physicochemical properties involved in ligand binding, forming a rich, combinatorial array of discrete data patterns (Keller and Vosshall 2016; Poivet et al. 2018; Barwich and Lloyd 2022). Traditional sensory models, designed for hierarchical and topographic data organization, prove inadequate for this task: If we adhere to these modeling principles, the olfactory system soon would be exhausted of its capacity to generate unique activity patterns. This critical aspect, though vital, has been largely overlooked by biologists but would be readily apparent to systems engineers accustomed to managing multidimensional data.

The question then is what functional information about odorants the proximal stimulus holds and how ORs encode and translate this information into neural signals. What precisely do these receptors detect? ORs are notoriously promiscuous, not monogamous; they engage combinatorially with a variety of physicochemical features, rather than responding to singular stimulus properties (Malnic et al. 1999).^2^ The combinatorial nature of these activations throws a wrench into any simplistic, one‐to‐one mapping we might hope to draw because ORs are *feature selective* but not *feature specific* (Barwich 2022).^3^ A single receptor can respond to diverse features across different odorants: It may react to the topological polar surface area of one odorant (O1), a functional group in another odorant (O2), and the specific ring size in yet another compound (O3). Additionally, the case of mixture perception, involving modulation mechanisms, further complicates this picture (Reddy et al. 2018; Xu et al. 2000; de March et al. 2020; Inagaki et al. 2020; Pfister et al. 2020; Zak et al. 2020; Barwich 2020a, 2021; Barwich and Xu 2021; Kurian et al. 2021; Xu et al. 2023).^4^ Given the inherent ambiguity in receptor coding, marked by feature‐underdetermined signals from its receptor, it is crucial to clarify what a glomerulus actually “represents.”

Briefly comparing olfaction and vision highlights how their functions are governed by distinct principles. Unlike the cells in the primary visual cortex, which selectively respond to specific orientations, olfactory glomeruli handle a broad spectrum of physicochemical features. Although ORs appear selective, this does not imply feature specificity; they respond to a broad range of physicochemical properties through combinatorial coding. This indicates a lack of a straightforward, predictable feature map within the bulb based on receptor activity. Thus, while the axonal convergence of olfactory sensory neurons (OSNs) into the glomeruli reduces stimulus dimensionality, the resulting input does not satisfy Mountcastle's criterion for cortical columns, as the neurons within a glomerulus are not activated by nearly identical peripheral receptive fields. Instead, viewed through an evolutionary lens, this combinatorial promiscuity must be understood in light of early mammalian diversification and the transition from aquatic to terrestrial environments, which triggered an explosion of OR genes and broadened their response tuning to adapt to a richer, more fluctuating, and less predictable chemical landscape (Yohe et al. 2020; Shepherd et al. 2021).

#### 3.1.2 The Argument From Development

Second, consider the *argument from development*: The organization of the olfactory bulb is strikingly flexible, defying the traditionally more rigid topographic model. Glomeruli form adaptively, not stereotypically, during development, shaped by both environmental and genetic factors. Studies tracking axonal connectivity and OR gene expression (Zou et al. 2009) emphasized this developmental plasticity, revealing olfactory organization as more fluid than static.

**FIGURE 4:**
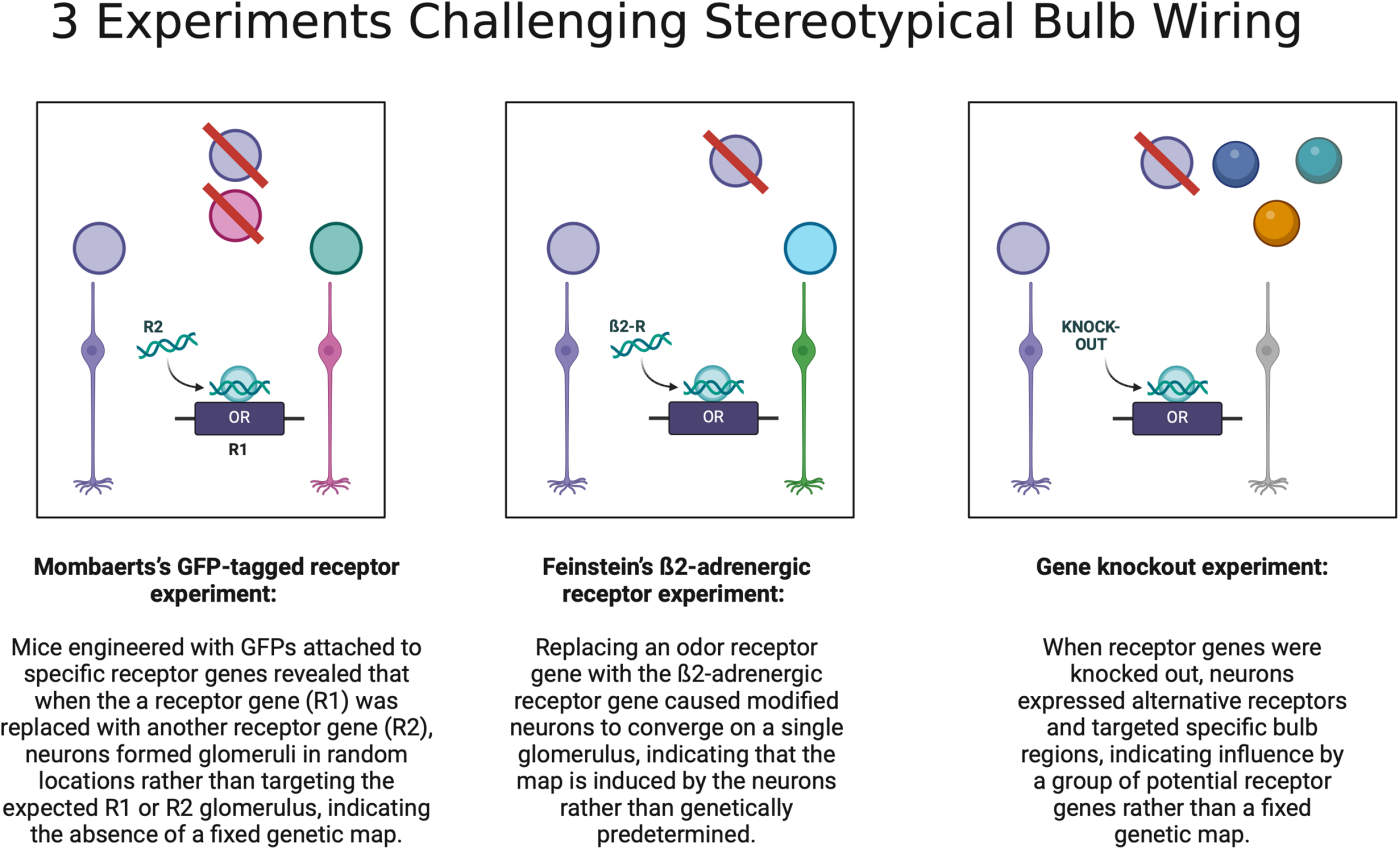
(Created in BioRender, Barwich 2025, https://BioRender.com/d22e637): Three experiments undermining stereotypic topography in the olfactory bulb, suggesting the olfactory map is dynamically organized by neurons rather than genetically prewired (in Feinstein et al. 2004; reviews in Zou et al. 2009; Barwich 2020a, Ch. 7).

Specifically, a series of interconnected studies on sensory neurons involving various substitutions and modifications of receptor genes demonstrates that the organization of glomeruli within the olfactory bulb is not as rigid and uniform as previously thought (Figure 4). For example, by replacing one receptor gene with another—such as swapping the mOR23 receptor gene (OlFR16) into the spot of the m71 receptor gene (OlFR151)—we see the creation of cells that send their axons to a different glomerulus than those expressing the original m71 or mOR2310 receptors. Feinstein and Mombaerts (2004) modified mice by linking GFP with OR receptor genes to see if neurons with altered genes would still target the mOR23 or m71 glomerulus, supporting the hypothesis that receptors guide axons to their genetic targets. Contrary to expectations, these modified neurons formed new glomeruli in unexpected locations instead of converging on the parental OR glomeruli. Further experiments replaced OR genes with nonolfactory ones, such as a β‐adrenergic receptor (Feinstein et al. 2004), or knocked out receptor genes entirely in some neurons (review: Zou et al. 2009; context: Barwich 2020a, Ch. 7).

The developmental wiring of the olfactory bulb diverges sharply from that of the visual system. Whereas vision depends on precise topographic maps, with specific features like orientation and spatial position systematically represented by selective cells, the olfactory bulb lacks such organized mapping. Although structured activity patterns in the bulb initially suggested the presence of a “chemotopic” or “odotopic” organization linked to distinct physicochemical features (e.g., in honeybees: Carcaud et al. 2018), this interpretation requires critical reexamination (especially in mammals).^5^ When viewed through the lens of genetic and developmental mechanisms, olfactory processing emerges as a distributed, overlapping network of activity rather than a neatly ordered map. This distinction is further supported by developmental studies (Zou et al. 2004), which reveal that glomeruli do not form through fixed, stereotyped patterns but instead arise from flexible, experience‐dependent processes.

In olfaction, the precise functional mapping from receptor cells to specific locations in the olfactory bulb remains elusive. Although there is a clear link between OSN receptors and their axonal destinations in the glomeruli, this pattern might not directly correspond to a functional representation of olfactory features. Rather than modeling glomeruli as some sort of target of OSN axons, glomeruli are better understood as the realization of OSN axons with like receptors coalescing and forming a glomerulus. In other words, the glomeruli are not there waiting for axons to find them. The axons make a glomerulus and thus constitute a developmental solution to the complex problem of connecting thousands of axonal populations to the brain. Such developmental arrangement might require a reappraisal considering its own functional significance. This insight aligns with the many philosophical challenges to the traditional biological and neuroscience tenet that structure dictates function (Allen et al. 1998). This tenet often fails under closer evolutionary examination, particularly considering phenomena like exaptation, where features developed for one purpose are co‐opted for another (Gould 1985). History in biological sciences consistently shows that many structures initially perceived as functional are byproducts of evolutionarily determined developmental necessities (Gould and Lewontin 2020 [1979]).

#### 3.1.3 The Argument From Circuitry

Finally, consider the *argument from circuitry*: Computational models of sensory encoding that emphasize topographic feature integration, tracking olfactory information from OSNs to the piriform, often reduce the bulb's sophisticated local circuitry to a mere byproduct of cellular complexity, treating it as a black box en route to odor feature mapping. In the face of the bulb's vast cellular diversity—including dense networks of periglomerular, juxtaglomerular, external tufted, mitral, and granule cells—it is easy to lose sight of the functional significance embedded within this delicate design.^6^ Viewed from a distance, the olfactory bulb's complex cytoarchitecture, with 15 known synaptic interactions forming both excitatory and inhibitory connections within a single glomerulus (Shepherd et al. 2021), is easily treated as mere mechanistic detail serving some end goal of “odor representation.” But this view may miss the point.

Odor images are better compared to faces than to objects such as tables and chairs (Shepherd 2012). While faces are made up of parts—eyes, noses, and mouths—we do not expect the fusiform face area (FFA) to map nostrils or iris color topographically. We recognize faces as wholes, shaped by context and the relationships between features, not by isolated parts. The Thatcher effect illustrates this, showing how face perception defies simple, hierarchical feature integration. That is not to say we cannot decompose faces or odors into their elements. Experts like painters and perfumers excel at exactly that, dissecting complex patterns with remarkable precision (Barwich 2017; Smith 2019). Nonetheless, this analytical skill is learned and not necessarily the brain's default mode (Churchland 2013; Barwich 2020a). Crucially, the parallel between faces and odors is more than phenomenological and reflects how the brain processes them at the neural level. Unlike early visual processing in its reliance on topographic maps, the olfactory bulb operates without comparable spatial constraints via its combinatorial code, processing odors through unique activation patterns across multiple glomeruli. This appears to mirror how the FFA processes faces by recognizing relational patterns, not by mapping features to specific regions. In both systems, recognition emerges from distributed networks that integrate contextual and relational information.

The olfactory bulb is not simply a sensory relay. It functions as an *active* early‐stage pattern discriminator and organizer (Kay and Murray Sherman 2007), perhaps more comparable to mid‐level visual areas like V2 and V4, where holistic object recognition takes place. Both face recognition and odor recognition rely on distributed neural circuits for fine‐grained pattern discrimination. Remarkably, this complexity does not require the six‐layered neocortex found in higher vertebrates. Complex pattern recognition is handled effectively by three‐layer cortical structures, the evolutionary precursors to the neocortex, supported by feedback and lateral mechanisms (both inhibitory and excitatory) that drive dynamic processing. For instance, fish with a three‐layer cortex perform sophisticated facial discrimination tasks, challenging the premise that a six‐layer neocortex is necessary for complex perception (Newport et al. 2016). Thus, its functional role and computational complexity reveal that the bulb serves as a hybrid processor, integrating primary sensory encoding, pattern discrimination, and associative functions.

Bulbar circuitry is remarkably sophisticated. This complexity extends beyond what can be discussed here, further encompassing a dizzying connectivity and functional integration with the anterior olfactory nucleus (AON), amygdala, and various neuromodulatory systems. Force‐fitting such a *polycomputational network* to a simplistic topographic model of glomerular activity patterns misses the point and undermines our efforts to understand how the brain truly operates.^7^ The unique architecture of the olfactory bulb defies orthodox sensory hierarchy models, instead offering a powerful lens into how complex perception arises from evolutionarily older neural circuits (Shepherd et al. 2021).

Ultimately, these insights on glomerular tuning, developmental wiring, and local circuitry shift our understanding of bulbar activity patterns from a deterministic, principally stimulus‐driven perspective to a more probabilistic developmental perspective on the neural architecture and information processing in olfaction (Cleland and Sethupathy 2006). Once we stop treating bulbar circuitry as just molecular machinery for representational ends, its intricate microcircuitry reveals something far more compelling: a dynamic, flexible system that offers a deeper, more powerful model of odor encoding (as discussed in Section 5.2).

### 3.2 Representational Drift in the Piriform Cortex

When placed alongside received models of the visual and auditory systems, olfaction presents a contrast. While the former systems show a degree of organizational consistency and structure in their sensory mappings, olfaction eschews this approach, favoring a more fluid and less predictable strategy (Barwich 2020a). This plasticity in neural encoding becomes most apparent within the piriform cortex (Barwich and Severino 2023).

**FIGURE 5:**
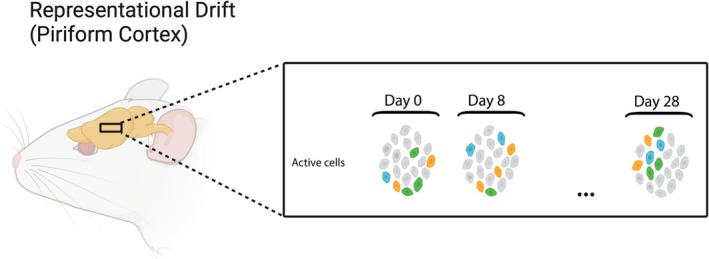
(Created in BioRender, Barwich 2025, https://BioRender.com/j57d807)): Representational drift in the piriform cortex. Neural representations of sensory information change over time, with cell responses gradually shifting, challenging the expectation of stable neural representation, even when the external stimulus remains constant. This change, driven by plasticity in neural circuits, can affect the stability and reliability of sensory perception and memory (image in box: Barwich and Severino 2023).

The piriform cortex, far from presenting an orderly neural territory, showcases its complexity through what appears to be a non–target‐driven domain (Stettler and Axel 2009; Sosulski et al. 2011; Chen et al. 2014; Diodato et al. 2016; Roland et al. 2017). Conventional sensory maps, like those observed in vision and hearing, depict a structured and consistent correlation between the external world and neural representations. In contrast, the organization—or conspicuous disorganization—of the piriform cortex reflects a plastic strategy finely attuned to the unpredictable dynamics of olfactory stimuli, tailored to a sensory environment marked by a high degree of molecular diversity and environmental variability. Variables such as air currents and humidity significantly influence how odors are perceived and processed (Philpott et al. 2004), necessitating a system that prioritizes flexibility over rigid mappings. Despite the seemingly random distribution of axonal projections (Figure 5, left), the piriform cortex adeptly synthesizes signals from various brain areas, forming variable activity patterns throughout olfactory information processing (Cohen et al. 2015; Wilson and Barkai 2018; Li and Wilson 2024). It emerges as a pivotal association hub (Haberly and Bower 1984, 1989; Hasselmo et al. 1990), linking memory, emotion, and decision‐making, thereby illustrating a sophisticated dynamic interplay that underpins its role in sensory processing.

A striking aspect of the piriform's functionality is its exhibition of representational drift (Schoonover et al. 2021). Representational drift is a phenomenon where neural responses to the same stimuli change over time, with cell responses gradually shifting (Driscoll et al. 2022; Rule et al. 2019), defying the expectation of stable neural representation. This finding not only is significant for our understanding of the olfactory system but also challenges the broader modeling assumption about sensory systems positing that neural responses are inherently stable and ought to compute stable and preferably spatial activity patterns as “representations” of perceptual features, states, or objects (Patel et al. 2014). Moreover, this manifestation of representational drift as neural plasticity, once primarily noted in regions like the hippocampus and posterior parietal cortex (Kentros et al. 2004; Lee et al. 2020; Rubin et al. 2015; Driscoll et al. 2017), was initially thought to be a marker of cognitive systems.^8^ However, representational drift is now documented in primary sensory cortices as well.^9^ This expansion of our understanding of where and how plasticity occurs suggests a broader, more dynamic capacity for modification in neural structures than previously appreciated.

The piriform cortex demonstrates an extraordinarily high degree of impermanence and responsiveness, adjusting its neural codes even in the face of repeated and consistent stimuli. Schoonover et al. (2021) demonstrated that the piriform's response to odorants is neither static nor fixed but dynamically changes over time, and it does so with startling rapidity. Within just 1 month, piriform “representations” can undergo profound transformations (Figure 5, right). Imagine the neural representation of an odor, “O1,” in early January. By the onset of February, its neural “representation” (i.e., its activity pattern in neural populations) has altered so drastically that it is as distinct from its January version as it would be from an entirely different odorant “O2.” Crucially, this neural drift continues unabated even with repeated exposure to the same odorant, rigorous daily stimulus training, or even fear conditioning, all of which have minimal effect on curbing the drift (involving its temporal delay without inducing representational stability).

In summarizing this section, it transpires that the topographic paradigm has markedly delayed, if not hindered, our understanding of olfaction by not adequately capturing the sophisticated neural dynamics involved in its signal processing. Recent observations from the piriform cortex challenge our fundamental understanding of neural organization and further prompt us to reexamine “representation” (Barwich and Severino 2023), a concept rooted in topographical thinking that has been widely adopted in neuroscience without adequate scrutiny of its causal explanatory value (Chemero 2000) or consistent theoretical interpretation among researchers (Favela and Machery 2023). Therefore, it is worth considering that topography may not be a foundational principle of neural organization but rather a contingent property emerging from the operations of specific systems, influenced by the affordances of stimuli or the functions unique to each sensory modality. This line of inquiry challenges a number of prevailing paradigms in neuroscience and suggests a shift toward understanding the brain as a highly flexible, context‐dependent, and associative processing hub. While this idea has been suggested in past decades, it has never gained the same level of traction or authority as the topographic model.

Thus far, we have explored how the persistence of the topographic paradigm is supported by historical developments and methodological convenience (Section 2). Recent advances in sensory neuroscience, particularly in the study of olfaction, are beginning to dismantle the characteristics of the topographic paradigm from which causal inferences have traditionally been drawn (Section 3). Beyond this corrective value, we now transition to the second part of our argument, introducing the positive heuristics that arise from incorporating the study of olfaction into broader neuroscience modeling (Sections 4 and 5). The remainder of this paper demonstrates that philosophical perspectives on science can be more than just critical—they can be constructive when used complementarily.

## 4 Philosophical Considerations: Olfaction, a Model for Neuroscience or “the Odd One Out”?

How can a stronger focus on olfaction, and recent insights into its processing, reshape and effectively benefit our approach to neuroscience? Historically, olfaction was routinely dismissed as an eccentric outlier within sensory systems, perceived as offering little of general neuroscientific insight. This perception contributed to its marginalization in terms of funding and attention throughout the 20th century (Barwich 2020a). However, the landscape of olfactory research transformed dramatically with Buck and Axel's (1991) groundbreaking identification of OR genes, revealing the largest family of G‐protein–coupled receptors, GPCRs in short, in the mammalian genome (Buck 2004, 2005; Axel 2005; Firestein et al. 2014; Barwich 2020b, 2021). This discovery not only integrated olfaction into the core of neurobiology and genetics but also highlighted its potential as a distinctive model for both pharmacology and computational as well as molecular neuroscience (Shepherd 1991; Barwich 2016). Considering these advances, it is thus surprising that olfaction remains largely undervalued, with its contributions and significance still not fully acknowledged within mainstream neuroscientific modeling and science education. This oversight exemplifies a broader issue in the field, where entrenched views can obscure emerging insights and hinder the recognition of valuable research avenues.

Indeed, olfaction continues to face a paradoxical dilemma, a catch‐22. On one hand, if smell is viewed as *too* analogous to vision, skeptics argue that it offers little new, given the advanced state of vision science. On the other hand, if it is considered *too* distinct from vision, critics claim it lacks broader relevance, confined to illuminating only its peculiar mechanisms. This predicament raises a pivotal question: What is the value of spotlighting and embracing olfaction as a model system in broader neuroscience? This issue highlights a core tension within scientific paradigms: the interplay between the quest for new knowledge and the assimilation of this knowledge within established scientific frameworks. Olfaction, residing at the border of similarity and distinctiveness, compels us to reflect on our conventional notions of what makes a scientific model valuable and relevant and to reconsider the criteria by which scientific utility is judged.

We offer two strategies to navigate this dilemma, beginning with an endorsement of *scientific pluralism*. Not long ago, philosophers of science offered a vision of science that discovered lawful generalities whose power was directly related to their ability to offer unifying explanations over a wide domain of phenomena (Cat 2024). As philosophers began to consider biology and even physics more carefully, their visions of a unified science began to give way to a more complex account of science where piecemeal integration replaced universalizing unification (Giere 1988, 2006). This pluralistic vision of science does not seek or expect single answers. Grounded in an appreciation for the multiple methodologies of science, the diverse perspectives of scientists, and the incredible variety within and between species in the biological sciences, pluralist accounts of science lead us to expect and value conflicting models whose results may not be easily reconciled (Feyerabend 2020; Cartwright 1999; Kellert et al. 2006; Wimsatt 2007; Mitchell 2003; Chang 2012). Importantly, pluralism does not suggest that these diverging approaches lack scientific robustness or fail to accurately account for real properties or causes in the world (Dupré 1993; Massimi 2022). Often, the discernment of structural and causally relevant features and relations depends significantly on the conceptual framework employed (Barwich 2013). For example, the classification of chlorine and its isotopes as one kind or several, based on either their electronic or nuclear structure, varies with the context of inquiry (Barnes 1982). When applied to neuroscience, the use of multiple model systems for building sensory processing models broadens our understanding of the phenomena at hand—regardless of how similar or dissimilar olfaction may be to vision.

Alongside embracing scientific pluralism, another response is to mind *the evolution of scientific paradigms*, considering how precisely the blend of similarities and differences across various model systems drives theoretical and empirical advancements in understanding scientific phenomena. Indeed, the history of olfactory research itself demonstrates the value of maintaining model and method pluralism. Prior to Buck and Axel's breakthrough discovery of olfactory receptors, Walter Freeman and colleagues had pioneered a sophisticated dynamical systems approach to understanding olfactory processing, developing some of the earliest mathematical models of neural population dynamics in sensory processing (Skarda and Freeman 1987; Kay 2018). Yet the influx of mainstream neuroscientists following the receptor discovery, while tremendously valuable in many ways, inadvertently led to these theoretical frameworks being sidelined in favor of more conventional paradigms derived from vision research. Ironically, these earlier dynamical system approaches are now experiencing a renaissance as neuroscience grapples with phenomena like representational drift (Barwich and Severino 2023). This historical trajectory, where a valuable theoretical framework was temporarily marginalized by the dominant paradigm only to resurface later as crucial for understanding neural dynamics, provides yet another compelling argument for maintaining theoretical pluralism in neuroscience.

The 20th century established vision as a dominant paradigm for studying both sensory information processing and broader brain functions (Shepherd 2009). However, vision might actually be more of an anomaly than commonly acknowledged.

First, take its role as a sensory paradigm: Vision's specific neural organization is intricately tailored to spatial navigation, decoding information from its predictable stimulus. Other senses like touch and olfaction, however, have evolved to respond to unpredictable stimuli, such as airborne volatiles in olfaction, which are influenced by complex fluid dynamics. Unlike vision, most sensory systems handle regularity without predictability, such as interoception (de Vignemont 2023), which relies on maintaining physiological equilibrium. Exploring the diversity among the senses reveals more than varying evolutionary paths shaped by distinct body–environment interactions, thereby offering a more granular view of neural functionality and its adaptive strategies. It also emphasizes the significance of embracing a “task‐ontology” approach in neuroscience (Burnston 2021; Nau et al. 2024)—a perspective that sheds light on how differences among sensory systems reveal diverse behavioral adaptations and elucidate the brain's underlying mechanisms for executing these varied functions.

Next, consider vision's role as a paradigm for general brain function: The profound influence of vision in shaping the history of neuroscience, becoming closely tied to specific modeling paradigms, may now be to its detriment—especially as the demand for alternative models is growing. To be sure, highlighting alternative systems, such as olfaction, does not imply that no unified causal principle might exist between these systems. Instead, it suggests that alternative systems help us move beyond our current conceptual blind spots, offering fresh perspectives on traditional paradigms. In this context, olfaction emerges as an ideal candidate for such an alternative.

Recent discoveries in olfaction, especially representational drift—with the subsequent discovery of similar phenomena in parts of the visual cortex (see Footnote 8)—challenge the traditional view that neural activity patterns remain stable over time. These insights into neural drift suggest a dynamic and non‐static nature of brain function, prompting a reevaluation of how we understand sensory, motor, and cognitive processes (Micou and O'Leary 2023). This dynamic processing exemplified by olfaction, particularly in how it combines external and internal sensory information, positions it as a powerful model for exploring “embodied” theories of perception and cognition (Chemero 2011; Crippen and Schulkin 2020). It highlights the need to shift from models that prioritize static representations to those that accommodate the dynamic nature of neural processes in brain–body–environment interactions.

Consequently, while vision has historically served as a foundational model, the evolving understanding of neural plasticity and dynamic information processing—evident in olfaction—underscores the potential of this sensory system to model broader, more generalizable neural mechanisms. Notably, this shift aligns with an increasing 21st‐century focus on neuroplasticity and challenges traditional notions of static neural representations by suggesting, for example, that alternative modeling frameworks such as predictive coding and dynamic systems theory offer valuable new perspectives in neuroscience (Barwich 2018; Barwich and Severino 2023). Insights gained from an alternative model system like olfaction thus can either bolster a general model of neural processing or prompt a critical reevaluation of the prevailing theoretical framework or some of its central premises.

The broader issue that we stress in this section is thus the question of why vision has dominated brain models for 60 years when olfaction offers an alternative model with its own distinct advantages. These considerations set the stage for examining a concrete alternative to vision‐based topographic mapping, one that builds on the distinctive characteristics of olfaction while offering broader insights into neural processing.

## 5 Application in Scientific Modeling: Transient Information Patterning and Memory Encoding Without Spatial Representation

Advances in understanding the genetic and developmental architecture of the olfactory system provide a promising framework for examining sensory processing, particularly the molecular mechanisms underlying dynamic sensory memory encoding. While the olfactory system's genetics warrant detailed investigation (Keller and Vosshall 2008), we specifically center on genetic transcription mechanisms and their role in neural plasticity. By analyzing how transcriptional processes influence information processing dynamics in olfaction, we outline a genetics‐based framework that could transform our understanding of both olfactory function and broader principles of neural signal processing.

### 5.1 Genetics: Perceptual Variation and Experience‐Dependent Modulation

Genetic transcription, which regulates gene expression, is crucial for cell functionality, differentiation, and environmental adaptability, offering a unifying perspective on how cellular processes undergird the dynamic encoding of environmental information. Over the past 15 years, and particularly in the last decade, transcription mechanisms have gained attention in neuroscience, especially in olfaction research (Ignatieva et al. 2014; Olender et al. 2016; Segura et al. 2022). In the brain, transcription mechanisms modulate neural processing, adapt brain functions to new experiences, maintain cognitive functions, and respond to environmental changes. The genetic basis of the olfactory system explains many of its more perplexing features, including its operational detachment from a topographically organized cortex and the strikingly personal variations in odor perception observed among individuals.

To examine how odor encoding is structured, kept flexible while stabilized by transcription mechanisms, we highlight three key genetic characteristics of the olfactory system. First, the olfactory system exhibits remarkable heterogeneity, with each person's OSNs expressing a unique set of ORs. We point to the genetic diversity of the olfactory system to illustrate how incorporating the genetic features of a sensory system can instruct “higher level” models of perception, as this individual expression profile contributes to the diverse ways people perceive odors (Trimmer et al. 2019). Second, the olfactory system demonstrates experience‐dependent transcription plasticity, which modulates OSN activity in response to different environments (Tsukahara et al. 2021). This plasticity allows the system to adjust dynamically to new and varying chemical stimuli, enabling responsive sensory processing and transient sensory memory encoding. Third, different OSN subtypes respond uniquely to sensory experiences, resulting in individualized transcriptional profiles (Tepe et al. 2018). Each neuronal subtype processes and adapts to sensory information in a distinct way, driven by its unique genetic transcription profile. Collectively, this creates a personalized receptor response repertoire in our noses, allowing individuals to perceive their environment in a remarkably tailored way.

First is *genetic heterogeneity*: The human olfactory system is highly heterogeneous and diverse across ethnic populations and individuals (Logan 2014). The ~400 genes for ORs in the olfactory system exhibit significant genomic variation, contributing to unique personal receptor repertoires. Of the variation present, 20%–40% of the receptor repertoire is formed by heterozygous haplotypes, some of which may be maintained by balancing selection. More specifically, the genetic foundation of olfactory receptors in both humans and dogs is marked by evolutionary forces such as genetic drift, purifying selection, and balanced selection (Olender et al. 2013). This array of genetic variations in mammalian OR genes contributes to the diverse ways people perceive odors (Keller et al. 2007; Menashe et al. 2007; Lunde et al. 2012; Trimmer et al. 2019).^10^ Even a single OR gene change can alter odor perception, with loss‐of‐function variants often associated with decreased intensity.

The significance of genetic heterogeneity in olfaction has been overlooked, most likely due to the reliance on genetically homogeneous model organisms like mice and fruit flies. Studies of the human olfactory system show that predictions based on these models do not straightforwardly translate to genetically diverse humans. For example, in model mice, each OSN expresses one of 1100 ORs, and OSNs with the same receptor send their axons to about two of the 1800 glomeruli in the bulb, creating a 2:1 convergence ratio. “Wild‐type” humans, however, express around 400 ORs, with over 5500 glomeruli, resulting in a 16:1 convergence ratio. This divergence suggests that odor coding in humans may involve more glomeruli for a more detailed odor representation (Maresh et al. 2008). While the full implications are still debated, the genetic basis of the olfactory system is crucial for understanding how odors are encoded and perceived.

Second is *experience‐ and environment‐dependent neuronal activity*: Genetic diversity in OR expression is closely linked to individualized transcription networks in OSNs. Each OSN has a unique transcriptome defined by its expressed OR, with distinct clustering based on different ORs (Tepe et al. 2018). For example, OSNs expressing Olfr727 differ from those expressing Olfr728 or Olfr729. This highly specialized gene expression is directly tied to the specific OR each OSN expresses.

**FIGURE 6:**
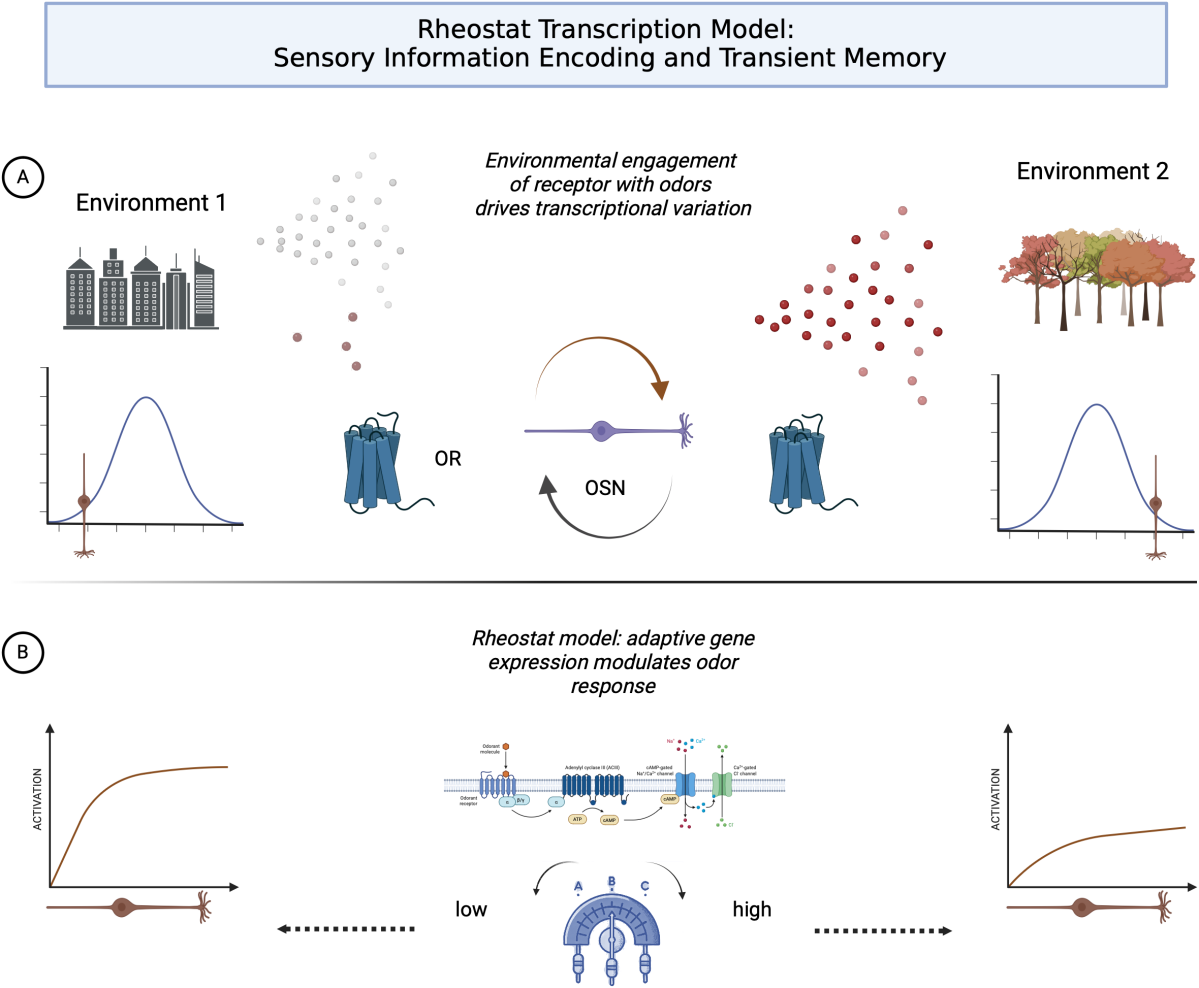
(Created in BioRender, Barwich 2025, https://BioRender.com/p80q802; modeled after Tsukahara et al. 2021): Transcriptional rheostat model. (A) Olfactory sensory neurons possess unique, odorant receptor–specific transcriptomes. Environmental odor engagement drives transcriptional variation, leading to adaptive changes in gene expression and odor responses. (B) The rheostat model suggests that adaptive gene expression modulates odor response. In vivo imaging shows that peripheral odor codes vary across environments. Olfactory sensory neurons adaptively shape their responses, distinguishing salient cues from predictable background.

These genetic transcription changes yield significant implications for our understanding of sensory encoding models. Tsukahara et al. (2021) demonstrated that OSN transcriptomes reflect environment‐dependent activity (or environmental states, henceforth: ES), which has significant implications for sensory encoding models. They showed that OSN activity adjusts to different chemical environments, with ES scores increasing or decreasing when OSN activity was artificially raised or lowered. In naturalistic odor environments, about 45% of OSN subtypes exhibited significant shifts in ES scores, indicating that specific odors engage distinct ORs differently. Based on these findings, Tsukahara et al. proposed a transcriptional “rheostat model” where gene expression adjusts sensory responses based on a neuron's history and current activity (Figure 6). Using single‐cell RNA sequencing (in mice), they revealed that each of the 1000 OSN subtypes has a unique transcriptome dictated by its OR, enabling unique responses to environmental odors. This diversity allows OSNs to adapt dynamically to changing sensory inputs, with over 70 genes modifying responses based on past exposures and environmental changes.

These transcriptional variations are organized and predictable based on OR–environment interactions, aligning with functional changes in odor processing and perception. By regulating genes that convert chemical signals into neuronal spikes, adaptive transcriptional responses in OSNs modify sensory perception, creating a form of transient “sensory memory” that influences future responses. Environmental changes drive gene expression adjustments in OSNs, preparing them for anticipated stimuli and encoding experiential learning at the cellular level. This supports a broader model where neurons customize their transcriptomes to efficiently handle expected environmental stimuli.

This *experience‐dependent genetic transcription plasticity* in the olfactory system differs from the more general experience‐dependent developmental plasticity and wiring observed in other senses, such as vision. Sensory neuron activity is modulated through transcription profiles to be environment dependent, meaning the genetic expression patterns within OSNs are dynamically adjusted based on environmental stimuli. As OSNs encounter different odors, their gene expression changes, tuning the neurons to be more or less sensitive to specific stimuli. This environment‐dependent modulation allows for a flexible and adaptive sensory system that can learn from past experiences and anticipate future stimuli, as well as *unlearn* these experiences, providing a form of short‐term and flexible sensory memory at the cellular level.

Third is *functional diversification* (at the cellular level): Research on OSN‐specific transcriptomes further shows that different neuronal subtypes are uniquely affected by sensory experiences. Genetic expression patterns in OSNs vary with sensory input, such that neurons in enriched environments exhibit different transcriptional changes than those in deprived settings. Each neuronal subtype processes and adapts to sensory information in a distinct way, driven by its individual genetic transcription profile. Thus, transcriptional mechanisms in OSNs significantly influence the functional diversity of neurons in the bulb. For instance, profiling transcriptomes from neurons at various stages of development and under different sensory conditions (naive, deprived, enriched), Tepe et al. (2018) uncovered developmental pathways and activity‐dependent changes in gene expression. These changes impact how neurons respond to new sensory inputs, linking external sensory experiences to internal genetic modifications and showcasing how external stimuli lead to internal cellular changes governing synaptic remodeling and circuit integration.

We suggest that these three features—genetic heterogeneity, experience and environment dependent transcription plasticity, and cellular functional diversity—contribute to the olfactory system's two most significant functional capabilities: its ability to recognize and respond to familiar odors and sensory backgrounds, while also being highly adaptive to new chemical environments and changing physicochemical stimulus combinations. In essence, different circumstances elicit varied sensory responses from individuals. The heterogeneous genetic makeup of the olfactory system, combined with its adaptability through OR‐specific transcription profiles, provides a versatile and adaptable causal framework for personalized and contextually tailored sensory responsiveness.

### 5.2 Morphological Computation: Sensory Encoding Sans Topography

The role of transcription mechanisms in modulating neural activity is often viewed as an adaptation process. For example, memory encoding activates specific transcription factors like CREB (cAMP response element‐binding protein), which facilitate the transcription of genes essential for neuronal functionality and plasticity. This activity‐dependent transcription, central to studies of long‐term potentiation (LTP) and long‐term depression (LTD), ensures that neuronal gene expression aligns with variations in neural activity patterns (Bickle and Barwich 2022). In olfaction, transcription mechanisms not only fulfill these general roles but also actively shape how information is encoded at the sensory periphery, particularly in OSNs.

Transcription mechanisms work alongside rapid biochemical adaptations to create a dynamic backdrop for information processing. These rapid adaptations include calcium‐dependent phosphorylation of ion channels, which can alter neuronal excitability within milliseconds; internalization of GPCRs, occurring within seconds to minutes; and modulation of second messenger cascades, particularly cAMP and IP3 pathways, which operate on a timescale of seconds. While these immediate responses occur through channel modulation and receptor changes, transcriptional changes generate a form of medium‐term peripheral olfactory memory encoding over hours to weeks. This multi‐timescale adaptation in OSN populations allows them to respond to both immediate fluctuations and persistent changes in their environment through a combination of rapid biochemical adjustments and “slower” (yet swift) transcriptome‐based rheostat adjustment of individual OSN activity. Thus, a significant portion of odor cognition is “offloaded” to computational processing at the periphery via both rapid biochemical adaptation and environmentally induced transcriptional changes.

Traditional views of neural processing often treat sensory systems as passive relays that merely transmit information to higher brain centers for computation. However, this perspective fails to account for the substantial processing that occurs at the sensory periphery itself. This limitation of traditional models becomes particularly evident in the olfactory system, where a plethora of high‐dimensional chemical information must be processed efficiently despite limited neural resources. The notion of *computational offloading* through morphological computation offers a more adequate and comprehensive framework for understanding sensory processing.^11^ Unlike traditional neural computation models that emphasize centralized processing in higher brain regions, morphological computation recognizes that an organism's physical structure and peripheral sensory systems can perform significant computational tasks without requiring explicit neural algorithms or additional processing steps and space. This distributed approach to computation proves especially relevant for understanding olfactory processing, where the physical and biochemical properties of OSNs themselves contribute to information processing. For example, in other sensory systems, the cochlea amplifies certain frequencies due to its shape, and the retina processes visual information such as edge and motion detection within the eye. These offloading mechanisms reduce the computational load for “higher” brain processing, allowing for more efficient and swift responses to environmental changes.

While these principles of morphological computation apply broadly across sensory systems, the olfactory system exhibits a particularly striking example through its capacity for continuous structural and functional modification. A distinctive feature that further exemplifies its fundamental operation via morphological computation is its robust capacity for adult neurogenesis, the generation of new neurons and synaptic connections throughout adulthood. While other sensory systems, such as vision, rely on both early development and ongoing experiential input for maintenance (Hubel and Wiesel 1963; Livingstone et al. 2017), the olfactory system stands apart through its continuous neuronal turnover. This process occurs in two key regions: the olfactory epithelium and the main olfactory bulb.

While evidence for ongoing lifelong adult neurogenesis in the epithelium remains contested across species,^12^ including humans, the process is well documented in the bulb (Whitman and Greer 2009; Brann and Firestein 2014). In the bulb, new neurons regularly integrate into existing circuits, contributing to enhanced learning, memory, and odor discrimination. Approximately 95% adult‐born cells differentiate into inhibitory adult‐born granule cells to form reciprocal dendrodendritic connections with the bulb's principal excitatory neurons: mitral and tufted cells. This continuous neural renewal, regulated by genetic programs and activity‐dependent transcription, provides a mechanistic bridge between molecular processes and systems‐level olfactory coding. Specifically, the interplay between genetic control of neurogenesis and experience‐dependent circuit modification helps explain how stable odor representations emerge at the population level in the bulb while allowing controlled representational drift in the piriform cortex, an essential feature for adaptive olfactory processing in dynamic chemical environments.

Recent evidence from computational modeling suggests that adult neurogenesis in the olfactory system serves a complex dual function in sensory processing. Using a spiking network model, Chen and Padmanabhan (2024) showed that while adult neurogenesis affects individual mitral and tufted cell responses in the main olfactory bulb, it preserves odor representations at the population level. The functional stability of glomerular input patterns represents a crucial mechanism for maintaining a consistent input processing stage despite ongoing neurogenesis. Each glomerulus receives input from OSNs expressing the same odorant receptor type, creating the appearance of a spatially organized map of odor responses (Section 3). This organizational principle ensures that even as individual OSNs are replaced through neurogenesis, the overall pattern of glomerular activation remains stable. Incoming OSNs are guided to their appropriate glomerular targets through molecular recognition mechanisms, maintaining the spatially patterned organization of odor information processing. Chen and Padmanabhan's (2024) findings further indicate how transcriptional regulation may shape circuit plasticity in this context. Their computational model illustrates how activity‐dependent transcriptional changes in adult‐born neurons may influence both local circuit properties and broader network dynamics.

Granule cell turnover via neurogenesis in the bulb further plays a key role in shaping piriform cortex dynamics by influencing multiple levels of circuit organization (Chen and Padmanabhan 2024). As described in Section 3, mitral/tufted (M/T) cells receive input from OSNs at glomeruli and send projections to the piriform in a randomized pattern, with each piriform cell integrating input from a unique subset of M/T cells. Granule cells, in turn, form reciprocal dendrodendritic synapses with M/T cells, modulating their activity (Shepherd et al. 2007). Recent in vivo imaging reveals that granule cells in the olfactory bulb maintain highly dynamic spines throughout their lifetime, with approximately 25% spine turnover occurring in less than 2 days (Sailor et al. 2016). While mature granule cell death is rare (with only a 0.66% daily decline after initial development), this structural plasticity creates continuous synaptic reorganization. Such ongoing remodeling affects M/T‐to‐granule, granule‐to‐granule, and piriform‐to‐granule feedback connections, creating what we term “dynamic stability”: a state where individual synaptic elements continuously reorganize while population‐level function is maintained.^13^ These shifts alter M/T firing rates, spike timing, and temporal response patterns, which then propagate to the piriform. Because piriform cells integrate inputs nonlinearly, even small shifts in M/T activity lead to widespread network reorganization. Some piriform cells increase in activity while others decrease, response durations shift, and the ensemble patterns encoding specific odors are reshaped. The result is continuous drift in piriform representations.

Adult neurogenesis in the main olfactory bulb emerges as a likely mechanism underlying olfactory plasticity, operating on timescales that match both representational drift in the piriform cortex and transcriptional changes in the periphery (Livneh and Mizrahi 2012). The continuous proliferation of granule cells responds dynamically to odor exposure (Shepherd et al. 2007), and Chen and Padmanabhan (2024) demonstrated that this drift is ecologically grounded: repeated odor exposure reduces drift through spike‐timing–dependent plasticity (STDP) at adult‐born granule cell synapses. When presynaptic and postsynaptic neurons fire in specific temporal patterns, they trigger calcium influx that activates distinct transcriptional programs, either strengthening or weakening synaptic connections. This creates a coordinated system where adult neurogenesis and STDP together bridge immediate sensory experience and longer‐term circuit adaptations, linking rapid electrical activity to the transcriptional changes that modify olfactory processing.

The relationship between transcriptional regulation and adult neurogenesis manifests through multiple mechanistic pathways. Transcription factors such as Sox2 and Pax6 directly regulate the proliferation and differentiation of neural progenitor cells in the olfactory epithelium. When new neurons integrate into existing circuits, STDP at their synapses triggers specific transcriptional programs through calcium‐dependent signaling cascades. These cascades activate immediate early genes within minutes, followed by structural and synaptic genes over hours, creating a molecular basis for long‐term circuit modification. This process exemplifies morphological computation in OSNs, as the physical properties of new synapses and their activity‐dependent modification contribute to information processing without requiring explicit computation by higher brain regions.

Such multilevel adaptation strategy in the olfactory system—combining rapid biochemical changes, transcriptional regulation, and ongoing neurogenesis—enables cells to adapt across multiple timescales (Table 1). The system can respond immediately to environmental changes through channel modulation, adjust hours to weeks through transcriptional mechanisms, and maintain long‐term adaptability through continuous circuit renewal. This experience‐dependent, multi‐timescale sensory adaptation for contextual encoding matches the ecological characteristics of chemical stimuli. Odorants frequently appear in varying chemical contexts, each altering their behavioral significance and perceptual interpretation. For example, indole in fecal plumes signals “contaminant,” whereas the same compound in coffee aroma does not carry the same implication. Hence, this encoding of olfactory information facilitates a dynamic sensory memory without topographic “representations” of stimulus features or perceptual objects.

**TABLE 1:** Temporal multilevel scaling of odor encoding in the olfactory system.

Timescale	Mechanisms	Functional role
Rapid or short term (milliseconds to seconds)	‐ Biochemical adaptations (e.g., calcium‐dependent phosphorylation of ion channels)	‐ Adjusts OSN sensitivity and responsiveness in real time
	‐ Internalization of G‐protein–coupled receptors (GPCRs)	‐ Enables immediate reactions to dynamic chemical environments
Medium term (minutes to hours)	‐ Activity‐dependent transcription mechanisms	‐ Fine‐tunes OSN responses to persistent odor stimuli
	‐ Calcium‐triggered activation of immediate early genes	‐ Creates a form of peripheral olfactory memory
	‐ Regulation of synaptic plasticity and receptor function	‐ Enhances odor detection and discrimination over time
Long term (days to months)	‐ Adult neurogenesis and circuit remodeling	‐ Maintains system plasticity and adaptability
	‐ OSN turnover in the olfactory epithelium	
	‐ Granule cell renewal in the olfactory bulb	‐ Ensures responsiveness to new odor environments
	‐ Transcriptional regulation of neuronal integration	‐ Supports long‐term adjustments in odor processing

**FIGURE 7:**
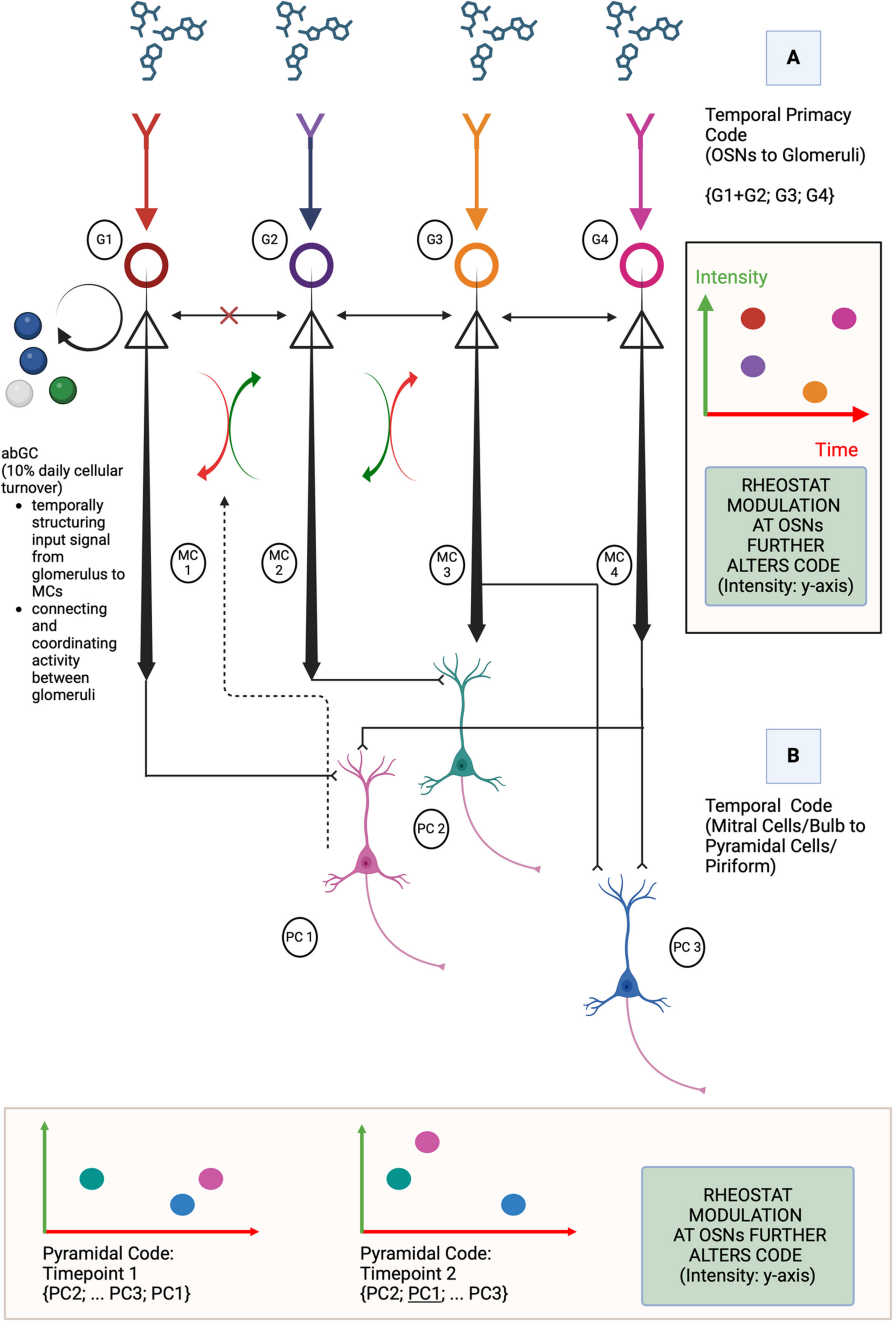
(Created in BioRender, Barwich 2025, https://BioRender.com/j95a045): Idealized temporal olfactory information code. Odorants bind to olfactory sensory neuron (OSN) receptors, with signals converging onto distinct glomeruli (G1–G4). (A) The glomerular code is structured along two dimensions: (1) temporal primacy, wherein receptor activation follows a combinatorial temporal sequence (Wilson et al. 2017), and (2) intensity modulation, governed by transcriptional mechanisms in accordance with the rheostat model (not depicted here). Mitral cells (MC1–MC4) receive input from glomeruli and project to pyramidal cells in the piriform cortex. This input is further modulated by granule cells (GCs), which shape and coordinate activity across glomeruli. For instance, in the schematic, G1/G2 are linked via inhibitory GC connections, while G2/G3 and G3/G4 exhibit excitatory interactions. Pyramidal cells in the piriform further feed back to GCs. Note that while the glomerular input pattern remains relatively stable due to OSN receptor specificity, mitral cell responses undergo experience‐dependent changes through continuous GC synaptic remodeling. This creates dynamic stability where population‐level odor codes maintain functional coherence despite individual cell variability. (B) The piriform code also operates along two dimensions: (1) temporality, shaped by GC‐mediated modulation of mitral cell input, and (2) intensity, adjusted through the rheostat mechanism and GC‐modulated input. Over time, the piriform code undergoes “representational drift” due to the turnover of GCs (while the precise turnover is unknown, Chen and Padmanabhan's (2024) model operated with a ~10% daily rate), dynamically altering connectivity patterns. For example, between timepoints, GC‐mediated inhibition between G1/G2 may disappear, while excitatory coupling between G2/G3 and G3/G4 weakens, leading to reduced synchrony and intensity. This shift propagates to pyramidal cell activation patterns, modifying both temporal order and intensity at the population level.

Our analysis of genetic and developmental features in the olfactory system reveals how sensory processing can operate without traditional spatial activity patterns. The olfactory bulb exemplifies “dynamic stability”: Individual mitral cells alter their odor selectivity over days in response to experience and behavioral associations (Pager 1983; Kay and Laurent 1999; Rinberg et al. 2006; Mandairon et al. 2006), yet population‐level “representations” remain functionally coherent. This dynamic stability demonstrates how brains compute through physical properties of neural tissue. In the olfactory bulb, structural dynamics *are* the computation: Granule cells find appropriate mitral cell partners through activity‐dependent remodeling. With spine turnover maintaining only 60% stability at 2‐day intervals, synchronized firing promotes spine formation while asynchronous activity triggers elimination. This mechanism allows individual mitral cells to shift their selectivity while preserving population‐level odor coding. The piriform cortex, then, inherits this structured variability. Each piriform neuron integrates inputs from multiple mitral cells, so changes in mitral tuning create representational drift as a systematic updating that maintains computational function while enabling environmental flexibility. Recent modeling confirms that such structurally dynamic networks can process novel odors while maintaining stable representations (Sailor et al. 2016); and Figure 7 illustrates how these complementary processes generate distinct odor codes in each region. This differential plasticity, involving stable population coding with flexible individual elements, represents a fundamental organizational principle extended across parallel processing streams. Overall, these findings highlight how peripheral transcription mechanisms and bulbar neurogenesis create inherent dynamics in olfactory processing, operating within a broader system of distributed computations across the olfactory pathway, including the lateral olfactory tract and AON.^14^

How, then, does our analysis of olfaction extend to other sensory systems and broader neuroscience? These observations invite a reassessment of neural encoding across various sensory systems. The olfactory system's parallels as well as divergences from vision and other sensory systems highlight the importance of exploring alternative model systems. Such exploration can uncover underlying causal relationships and processes that might otherwise remain hidden.

## 6 Discussion

This paper challenged the dominance of topographic models in neuroscience that map the brain as structured three‐dimensional spaces reflecting the external world. Despite its historical utility, mounting evidence suggests that neither columnar organization nor static neural maps are universal features of sensory cortices. We argued that rigid adherence to this model has constrained our exploration of alternative frameworks and limited our understanding of neural functions.

It is crucial to recognize that sensory perceptions across different modalities do not necessarily arise from similar causal processes. Unless one assumes that sensory processes share an evolutionary or developmental origin, differences among sensory processes should be expected. Adequate models must map onto both causal processes and their outcomes without presupposing a unitary model. The marked differences between olfaction and vision, for example, caution against generalizing the topographical model across all sensory modalities. Our analysis of genetic studies of the olfactory system, as well as the recent discovery of “representational drift” in the piriform cortex, demonstrates that traditional models of sensory encoding, relying on static topographic maps, do not adequately capture the dynamic and flexible nature of sensory information processing.

Our concern about the term “representational drift” echoes broader critiques of representational language in neuroscience. As Guest and Martin (2025) argued in their analysis of the “cognitive map” construct, neurocognitive theories that assume stable, map‐like representations suffer from both infinite regress and computational inertness. Accordingly, when scientists focus on correlational matches between external structures and neural data (the “content”), they neglect to specify the computational mechanisms (the “vehicle”) that would actually explain cognitive capacities. What has been termed “representational drift” in neural populations thus appears as a misnomer that signifies our observational perspective rather than biological reality. When we observe changes in population‐level neural activity, these inevitably manifest in individual neuronal responses. However, describing these changes as “drift” in “representations” imposes our interpretative framework onto what is more accurately described as a dynamic rearrangement of responses among the neuronal population as a fundamental feature of neural processing rather than a deviation from a static norm. Research on drift as population‐level neural dynamics extends beyond the olfactory system to neocortical areas, as demonstrated in pioneering studies of the olfactory bulb (Freeman and Schneider 1982), piriform cortex (Barrie et al. 1996), and other cortical regions (Ohl et al. 2001).

This perspective becomes particularly compelling when considering how different environmental constraints shape sensory systems. While the visual systems of land mammals are optimized for spatial navigation in relatively stable environments, aquatic species navigate through more dynamic, three‐dimensional spaces where visual scenes are less predictable. Fish and marine mammals, for instance, cannot rely on unchanging visual landmarks for navigation (Jacobs 2012). Their visual systems might therefore employ more flexible coding strategies reminiscent of olfactory processing, suggesting that what we consider “typical” visual processing may actually represent a specialized adaptation to terrestrial environments rather than a universal principle of sensory organization. Therefore, our aim in reevaluating sensory processing models via olfaction is to emphasize the importance of exploring alternative mechanisms that have been largely neglected.

Acknowledging causal‐mechanistic differences between systems like vision and olfaction does not imply complete disunity in neural processing. Rather, it demonstrates the importance of deriving principles from the intrinsic characteristics of each system. This paper thus embraced scientific pluralism, not as a barrier to developing a more unified theory of sensory processing but as a foundational strategy for integrating diverse approaches and model systems. Pluralism is crucial for building a comprehensive theory across different sensory modalities. For example, our exploration of how genetic mechanisms structure information encoding in olfaction aligns with broader rule‐based accounts of sensory encoding from studies of the visual system. That said, by recognizing the distinct nature of each sensory system, we highlight the limitations of applying system‐specific principles universally, potentially mistaking the more idiosyncratic aspects of sensory processing for general principles of neural organization.

Consider two parallels to illustrate a broader shift toward rule‐based explanations in scientific models. First, consider the transformation from preformation theory to modern developmental biology (Maienschein 1997, 2000, 2005). Initially, preformation theory, supported by 17th‐century scientists like Nicolas Hartsoeker and Antonie van Leeuwenhoek, held that development involved merely enlarging a fully formed organism preexisting in the egg or sperm. This view was overturned in the 19th and 20th centuries as discoveries in cellular biology and embryonic development revealed that development is a dynamic, rule‐based process driven by genetic, cellular, and environmental instructions. This shift—from seeing development as predetermined and static to recognizing it as complex and responsive to genetic and environmental interactions—mirrors a similar change in neuroscience. Historically, some views of brain function analogously assumed a centralized “homunculus” that managed sensory data and decisions (Finger 2001; Oeser 2010 [1938]). Meanwhile, modern neuroscience understands the brain as a distributed network where various regions concurrently process inputs, integrate information, and influence outputs, moving away from homuncular interpretations (McClelland et al. 1987; Dennett 1993; Churchland 1995; Sporns 2016). Nevertheless, remnants of the homunculus idea persist in contemporary research on the brain's “wetware,” as seen in the topographic paradigm. Our call for a paradigm shift in sensory neuroscience thus reflects a broader transition toward understanding biological processes as rule governed and adaptable rather than fixed and predetermined.

Another analogy concerns GPS technology, which navigates and processes information through dynamic, rule‐based mechanisms rather than static spatial representations. GPS technology dynamically adjusts routes based on real‐time data, calculating precise locations using the timing of signals from a network of satellites, rather than relying on fixed spatial maps. This dynamic routing, responsive to current conditions such as traffic and road closures, demonstrates that effective navigation can be achieved through numerical and signal data, processed algorithmically without the need for a visual representation of geographical details. Similarly, in the brain, sensory information processing can operate through dynamic, rule‐based mechanisms that adapt to real‐time inputs and conditions. Both systems utilize basic sets of instructions to process information dynamically, without a central “reader” or homunculus, indicating a distributed, rule‐based approach to processing tailored to the specific needs and constraints of each system.

These analogies underline the central thesis of our paper: Scientific modeling has progressed from static, deterministic models to dynamic, rule‐based models in both biological processes and technological developments. This philosophical and practical transformation in neuroscience proposes that embracing a flexible, rule‐based approach to sensory processing will offer a more precise and adaptable framework for investigating a plurality of neural mechanisms of sensory and cognitive functions.

## Author Contributions

**Ann‐Sophie Barwich:** conceptualization, investigation, methodology, project administration, supervision, validation, visualization, writing – original draft. **Stuart J. Firestein:** conceptualization, investigation, methodology, supervision, validation, visualization, writing – original draft. **Michael R. Dietrich:** conceptualization, validation, writing – original draft.

## Conflicts of Interest

The authors declare no conflicts of interest.

## Peer Review

The peer review history for this article is available at https://www.webofscience.com/api/gateway/wos/peer‐review/10.1111/ejn.70224.

## Endnotes

^1^
Mombaerts (2004) subsequently reexamined this doctrine, proposing a different model that posits an initial developmental stage, characterized by oligogenic expression, which is subsequently refined through processes of positive and negative selection, ultimately leading to cells that typically express a single receptor.

^2^
See also two earlier studies (Firestein et al. 1993; Ma and Shepherd 2000).

^3^
Ross (2021) examined the concept of specificity in causal relationships, focusing on value specificity, which refers to the range of effects a single cause can produce. This means that while odor receptors are selective in their responses, they are not limited to responding to only one specific feature or type of feature.

^4^
Suggested allosteric modulation in odor mixture coding (Xu et al. 2020): For instance, receptor R1 may cease to respond to odorant O1 when it is accompanied by odorants O2 and O3, while receptor R2 may only respond to O2 in the presence of O1 and O3, yet not in isolation.

^5^
Odor receptors in mammals and insects are notably genetically distinct with varying evolutionary origins (Niimura 2009).

^6^
This dense neuronal composition routinely yields surprises, such as the discovery of short axon cells that may be the first type of interneuron connecting directly to the sensory input layer (Eyre et al. 2008).

^7^
“We provisionally define polycomputing as the ability of a material to provide the results of more than one computation in the same place at the same time” (Bongard and Levin 2023).

^8^
Although it remains equally unclear what defines such a “mark of the cognitive” (Allen 2017).

^9^
Following the findings of Schoonover et al., subsequent publications by Marks and Goard (2021), Deitch et al. (2021), and Bauer et al. (2024) uncovered comparable patterns of representational drift in the visual cortices of mice, specifically including the striatum (V1). While we observe notable differences in how this drift presents in the olfactory and visual systems—with the visual system maintaining representations (Keinath et al. 2022)—these discrepancies are not simply curiosities but warrant deeper examination. The varying levels of adaptability observed might be key to understanding how the brain processes sensory information differently across modalities.

^10^
Early investigations into the causal factors influencing individual variability in odor perception, particularly regarding androstenone, were conducted by Wysocki et al. (1989).

^11^
In cognitive science, the concept of cognitive offloading challenges the traditional view that cognition is confined to the brain, suggesting instead that cognitive processes extend into the body and environment (Hutchins 1995; Hendriks‐Jansen 1996; Clark 2013). This allows tasks like memory or computation to be offloaded onto external media, such as using paper for calculations or microscopes for scientific investigations. We also encounter this concept in autonomous robotics, where robots are engineered to leverage their physical structure to reduce the need for active control and computation (Brooks 1999; Beer 2003). For example, robots might utilize specific body shapes to simplify motion control or incorporate soft materials to adapt to various objects without relying on complex sensors.

^12^
The view of continuous neuronal regeneration throughout adulthood appears oversimplified when confronted with research suggesting that neurogenesis may function primarily as a compensatory mechanism responding to injury or disease (Carr and Farbman 1992, 1993). This insight derives from studies of the principal model organism in neuroscience—mice aged 30–60 days. Neurogenesis appears robust in young adults (30–60 days); however, it undergoes age‐dependent decline in proliferation density within the olfactory epithelium by 9 months of age (Weiler and Farbman 1997). Although several early studies suggested age‐related effects on neurogenesis, research largely overlooked age as a crucial variable. In an artificial Eden of a pathogen‐free environment, these neurons may persist for up to a year.

^13^
These spine turnover measurements from Sailor et al. (2016) likely vary considerably with age, behavioral context, and sensory experience. Thus, the functional impact of this structural plasticity may depend more on the continuous formation and elimination of synaptic connections than on cell turnover itself. Indeed, while one previously cited 10% daily granule cell turnover appears to reflect different phenomena (possibly neuroblast death during development or confusion with olfactory sensory neuron turnover in the epithelium) (Chen and Padmanabhan 2024), the critical computational feature may be the ongoing synaptic remodeling rather than cell replacement per se. Establishing definitive turnover rates remains challenging and will require tracking both spine dynamics and cell survival across varied experimental conditions.

^14^
This organizational principle of complementary dynamics extends to parallel processing streams that serve distinct computational roles. For example, recent research indicates that the largely understudied AON, receiving primarily tufted cell input from the bulb, specializes in odor identification and discrimination (Shepherd et al. 2021; McKissick et al. 2024). Meanwhile, the piriform, receiving predominantly mitral cell input and integrating AON projections, processes odor context and meaning. This functional interpretation is supported by the marked sensitivity of mitral cells to behavioral associations, environmental enrichment, and learned significance (Pager 1983; Bhalla and Bower 1997; Kay and Laurent 1999; Rinberg et al. 2006; Mandairon et al. 2006).
